# Spectrum of Gastrointestinal, Hepatobiliary, and Pancreatic Involvement in Hantavirus Infection: A Narrative Review

**DOI:** 10.7759/cureus.110352

**Published:** 2026-06-06

**Authors:** George S Zacharia, Mohammad A Masood, Muhammad H Ashraf, Saran Lal A Mokan Dasan, Fouad K Hocine, Misbahuddin Khaja

**Affiliations:** 1 Internal Medicine, BronxCare Health System, New York, USA

**Keywords:** gastrointestinal, hantavirus, hemorrhagic fever, liver, pancreatitis, renal failure

## Abstract

Hantavirus infection is a zoonotic, rodent-transmitted viral disease that presents with two distinct clinical syndromes: hemorrhagic fever with renal failure (HFRS) and hantavirus pulmonary syndrome (HPS). The 2026 cruise ship hantavirus outbreak has once again brought this infrequent disease into the limelight. Gastrointestinal (GI), hepatobiliary, and pancreatic manifestations, though frequent, have garnered less attention in patients with hantavirus infections. This narrative review summarizes the available literature on GI and hepatopancreatic involvement in hantavirus infection. A comprehensive review of the literature published between 2000 and May 2026 was conducted using PubMed. Eligible studies included observational studies, systematic reviews, meta-analyses, case series, and case reports describing GI, hepatic, biliary, or pancreatic manifestations in human hantavirus infection. The literature review identified GI symptoms among the earliest and most frequent prodromal manifestations of hantavirus disease. Nausea, vomiting, abdominal pain, and diarrhea often precede organ-specific complications by several days. GI bleeding develops as a component of hemorrhagic syndrome. Hepatic involvement most frequently manifests as transient elevations in aminotransferase levels and, less frequently, as hyperbilirubinemia. Gallbladder wall thickening, acalculous cholecystitis, instances of hantavirus-triggered autoimmune hepatitis, and acute pancreatitis have also been reported. Current management strategies are primarily supportive, including renal replacement therapy for severe renal injury and ventilatory support and extracorporeal membrane oxygenation for severe pulmonary disease. Antiviral therapies such as ribavirin and favipiravir have shown inconsistent or limited clinical benefits. Limited evidence links early and prominent GI symptoms and transaminitis with more severe disease and a worse prognosis. A high index of suspicion in patients with GI symptoms may facilitate earlier diagnosis and allow risk stratification. However, the current evidence is sparse, mostly derived from case reports, case series, and small retrospective studies, which limits our understanding of GI and hepatobiliary-pancreatic manifestations in patients with hantavirus infections. Large prospective studies are needed to clarify the mechanisms, incidence, and prognostic significance of these manifestations.

## Introduction and background

Hantavirus received significant global attention following the 2026 cruise ship outbreak. Historically, this rare zoonotic infection has been involved in at least two major outbreaks involving the US diaspora: the first outbreak during the Korean War, which involved nearly 3000 US troops, and the second outbreak in the Southwestern United States in the early 1990s [1]. The exact burden of the disease worldwide is not clearly quantified, but the World Health Organization estimates range from 10,000 to over 100,000 cases annually [2]. Nearly 90% of documented cases worldwide are reported from Asia, especially China [1]. Cases of infection caused by the hantavirus subtype Puumala virus are most frequently reported in Northern and Central Europe. In the West, cases are more frequent in South America, especially Argentina [2]. In the United States, according to the Centers for Disease Control and Prevention's 2023 estimates, 890 confirmed cases of hantavirus infection have been reported [3]. The outbreak that has captured attention of late developed on board a Netherlands-flagged cruise ship and involved 13 people: 11 confirmed and 2 probable cases, including 3 fatal cases (as of May 27, 2026) [4]. It is widely believed that the index case acquired the infection in South America, where he had been traveling for nearly three months prior to boarding the ship. Person-to-person transmission of the virus likely led to subsequent cases on board [5].

Rodents are natural reservoirs of infection and sources of human infection. The classical presentations include hemorrhagic fever, renal dysfunction, and pulmonary syndrome. Interestingly, hemorrhagic fever and renal dysfunction predominate in Asia and Europe, whereas cardiopulmonary syndrome predominates in the Americas [2]. The case fatality rate is approximately 50% in the Americas, while it is <15% in Asia and Europe; this likely reflects differences in the disease spectrum [2]. Diagnosis relies on antibody testing and viral nucleic acid polymerase chain reaction (PCR); however, it is often challenging because of the rarity of the disease and the overlap of its clinical presentation with other viral febrile illnesses. Currently, there is no specific treatment for hantavirus infection. This narrative review summarizes the published literature on gastrointestinal (GI), hepatobiliary, and pancreatic manifestations of hantavirus infection.

## Review

Methodology

This narrative review summarizes the current literature on GI, hepatobiliary, and pancreatic manifestations of hantavirus infection. Comprehensive PubMed searches were conducted to identify pertinent literature published from 2000 through May 2026. Original articles, observational studies, systematic reviews, meta-analyses, case series, and case reports describing GI, hepatobiliary, or pancreatic involvement in human hantavirus infection were considered eligible for inclusion. Experimental animal studies without direct clinical relevance were excluded, except for studies in the evolving literature on newer therapeutic modalities. The authors independently screened titles and abstracts for relevance, followed by a full-text review of the selected articles. Given the heterogeneity of the published literature and the predominance of case reports and small observational studies, a quantitative meta-analysis was not performed. Instead, data from the included studies were extracted, synthesized, and organized under the subheadings of virology; clinical manifestations-general, GI, hepatobiliary, and pancreatic; diagnosis; and treatment. References from the included studies and relevant review articles were also screened to obtain pertinent supplementary information. The narrative design of the review, unlike that of a systematic review, could have introduced selection bias, and some relevant studies may not have been identified or included.

Virology

Hantaviruses are single-stranded ribonucleic acid (RNA) viruses belonging to the family Hantaviridae and the order Bunyavirales [6]. Multiple strains of hantaviruses have been described; from a clinical perspective, they are classified as causing either hemorrhagic fever with renal syndrome (HFRS) or hantavirus pulmonary syndrome (HPS) (Table 1) [7-9]. The strains implicated in HFRS include Seoul virus, Hantaan virus, Puumala virus, and others, while Andes virus and Sin Nombre virus are responsible for most cases of HPS [8]. Rodents are the natural reservoirs; rodent species vary among hantavirus strains, e.g., the long-tailed pygmy rice rat (*Oligoryzomys longicaudatus*) for Andes virus, the deer mouse (*Peromyscus maniculatus*) for Sin Nombre virus, and the Norway rat (*Rattus norvegicus*) for Seoul virus [9].

**Table 1:** Hantaviruses causing human infections The  Sochi virus is a genetic variant of the Dobrava-Belgrade virus, reported from Russia [7]. HFRS: hemorrhagic fever renal failure syndrome; HPS: hantavirus pulmonary syndrome.

Viral Syndrome	Virus Variant	Geographic Distribution
HFRS	Hantaan	China, Korea, Russia
	Seoul	Worldwide
	Puumala	North Europe, Central Europe
	Dobrava-Belgrade	Balkans, Eastern Europe
	Saaremaa	Baltic, Eastern Europe
HPS	Andes	South America
	Sin Nombre	United States, Canada
	Laguna Negra	Bolivia, Paraguay
	Bayou	United States
	New York	United States
	Choclo	Panama

Human infections are typically acquired from infected rodents. Rodents shed the virus in urine, feces, and saliva. Transmission to humans most often occurs through contact with, or inhalation of, infected material and rarely through bites or scratches from infected rodents. Andes virus is the only known hantavirus documented to undergo human-to-human transmission, often through prolonged close contact [6].

The viral glycoproteins interact with host-cell surface integrin receptors. The best-described integrins that facilitate hantavirus entry into human cells are αIIaβ3 and αvβ3 in HFRS and HPS, respectively [9]. Once inside host cells, the viral nucleocapsids and RNA-dependent RNA polymerase are released, thereby facilitating infection and replication. Endothelial dysfunction and increased vascular permeability are key pathogenic mechanisms in hantavirus infections; however, the mechanisms that culminate in these manifestations remain unelucidated. It is widely believed that the virus itself is noncytopathic; hence, the lytic effects may be secondary to an overly robust or dysregulated host inflammatory response [10]. The renal medullary and pulmonary capillaries are primarily affected in HFRS and HPS, respectively; however, the mechanisms underlying these predilections remain unexplained [10].

Clinical manifestations

General

The prodrome of hantavirus infection is strikingly consistent across causative species. Fever and myalgia are virtually universal. In the landmark description of the first 17 confirmed HPS cases, both were present in 100% of patients, alongside headache in 71% and GI symptoms in 76% [11]. In HFRS, the prodrome is similarly characterized by abrupt-onset fever, severe headache, backache, myalgia, abdominal pain, facial flushing, and conjunctival injection, reflecting early endothelial activation and microvascular leakage [12]. This prodromal phase typically lasts three to seven days and is clinically indistinguishable from influenza, leptospirosis, dengue, and other viral febrile illnesses. A febrile illness accompanied by thrombocytopenia, regardless of age, should prompt consideration of hantavirus infection in endemic regions. Hemorrhagic manifestations are more prevalent than is commonly appreciated. In one Andes virus cohort, hemorrhage was evident in 81% of patients, with moderate-to-severe bleeding in 63% [13].

The two hantaviral syndromes diverge in their dominant organ involvement. In HFRS, acute kidney injury is the defining feature, with thrombocytopenia, hyponatremia, and urinary volume serving as the strongest indicators of severity [14]. In HPS, noncardiogenic pulmonary edema with myocardial depression has a case fatality rate of 35%-50% in the Americas, and HPS is frequently misdiagnosed at initial presentation [11,15]. Beyond these cardinal manifestations, GI, hepatobiliary, and pancreatic involvement are increasingly recognized as clinically significant complications of hantavirus infection and constitute the focus of this review.

GI Involvement

GI symptoms are among the earliest manifestations of hantavirus infection. Nausea, vomiting, abdominal pain, and diarrhea are reported as GI symptoms in hantavirus infection. These symptoms may precede the development of organ-specific features. Witkowski et al. used human intestinal Caco-2 monolayers and demonstrated that Puumala virus invades intestinal epithelial cells, disrupts tight junction proteins, and induces paracellular leakage [16]. These findings portray GI symptoms as a direct reflection of viral epithelial injury rather than a nonspecific prodromal feature. In a cross-sectional study from Turkey, diarrhea was a statistically significant discriminating feature between serologically confirmed and unconfirmed hantavirus cases [17].

At least one GI symptom was reported at admission among patients with Puumala virus infection [18]. In a cohort of 23 patients with confirmed HFRS, Christova et al. found abdominal pain and vomiting to be prominent manifestations among Dobrava-Belgrade virus-infected patients [19]. Kim et al. reported two cases of serologically confirmed HFRS in Korea in which acute diarrhea was the primary presenting complaint, with no obvious hemorrhage or renal impairment at the initial evaluation [20]. Cases of HFRS masquerading as an acute abdomen have been reported in the literature, with at least two cases undergoing appendectomy in the setting of hantavirus infection. In both cases, the appendix was pathologically normal; however, in one case, hantavirus was confirmed by polymerase chain reaction (PCR) and immunohistochemical staining of the resected appendix [21,22]. Early and prominent GI involvement has also been associated with greater overall disease severity in HFRS, with the most severe cases in one outbreak series demonstrating GI symptoms within one to two days of fever onset [7].

Luminal GI bleeding is an underrecognized complication of hantavirus infection, driven by the endothelial dysfunction and coagulopathy that underlie the viral hemorrhagic syndrome. Nuutinen et al. performed gastroscopy in 10 consecutive patients with HFRS and found hemorrhagic gastropathy in every case, with lesions more prominent in the proximal stomach and extending to the duodenum in 70% of patients [23]. Histological examination revealed edema in the lamina propria without inflammatory infiltrate, consistent with microvascular leakage rather than primary mucosal inflammation. Hemorrhagic lesions resolved completely on follow-up endoscopy three to eight weeks later, suggesting a self-limiting process tied to the acute vascular phase of infection [23]. In the meta-analysis of 140,295 HFRS patients, GI bleeding was significantly more prevalent among non-survivors than among survivors (OR: 2.784; 95% CI: 1.602-4.839, p < 0.001), establishing it as an independent marker of severe disease [24].

In the 1993 HPS outbreak in the Southwestern United States, GI symptoms were reported in up to 76% of patients. GI symptoms were prominent during the prodrome and often preceded respiratory compromise by several days [11]. Maleki et al., in a retrospective study of 93 Andes virus-infected HPS patients in Argentina, found that intestinal fatty acid-binding protein, a validated serum marker of intestinal epithelial damage, was independently associated with a fatal outcome by multivariate analysis (OR 1.64; 95% CI: 1.01-2.64) [25]. This provides objective serologic evidence that intestinal injury is a measurable component of HPS pathophysiology with direct prognostic implications.

Hepatobiliary Involvement

Hepatic involvement is a well-documented manifestation of hantavirus infection, with the most frequent finding being transient elevation of liver enzymes. Tervo et al., in a prospective study of 66 hospitalized patients with acute Puumala hantavirus infection, found that alanine aminotransferase was elevated in 36% of cases during the acute phase, with levels normalizing during convalescence. The change in transaminase levels was independent of ethanol consumption [18]. Elevated levels of alanine aminotransferase (ALT) and aspartate aminotransferase (AST) were reported among patients during the 1993 US outbreak; the admission mean values were 55 IU/mL and 112 IU/mL, respectively [11]. A cohort study from China, including 395 patients with HFRS, reported elevations in ALT and AST to >40 IU/mL in 77% and 88% of patients, respectively. An elevated total bilirubin level (>17.1 μmol/L; >1 mg/dL) was reported in 25% of cases [26]. Liver biopsies in patients with HFRS have revealed midzonal necrosis and mild mononuclear infiltrates [27].

Cases of hantavirus infection triggering autoimmune hepatitis have been reported in the literature, likely via a Th2 helper cell immune response [28,29]. Hantaan virus has also been postulated to have a role in unexplained acute hepatitis, negative for viral hepatitis A-E, Cytomegalovirus, Epstein-Barr virus, and drugs. However, in the study, hantavirus antibodies were detected in only 13 of the 83 unexplained acute hepatitis cases; the authors also failed to mention whether autoimmune hepatitis was excluded [30]. A recent study compared two rodent-borne human zoonoses: leptospirosis and hantavirus infection. It reported an indistinguishable initial clinical presentation, but renal and hepatic dysfunction were more common and more severe in patients with leptospirosis, although the difference did not reach statistical significance for most parameters [31].

Liver damage is reported to be significantly more prevalent among non-survivors with HFRS. Patients who died were more likely to have elevated AST and ALT, with standardized mean differences of 1.067 (95% CI: 0.645-1.490, p < 0.001) and 0.829 (95% CI: 0.258-0.936, p = 0.002), respectively [24]. Similarly, Chen et al. reported median AST (167 IU/mL vs. 79 IU/mL; p = 0.001) and ALT (76 IU/mL vs. 56 IU/mL; p = 0.017) levels that were significantly higher in patients who died than in those who survived the illness. However, in multivariable analysis, transaminase levels did not show a significant association with mortality [26].

Gallbladder involvement is also reported in patients with hantavirus disease. A retrospective study by Kim et al. identified gallbladder wall thickening of 4 mm or greater in 43% of cases with HFRS. Patients with wall thickening had lower platelet counts, lower albumin levels, higher transaminase levels, and higher rates of hemodialysis, ascites, and pleural effusion than those without thickening. All five patients who died during the study period had gallbladder wall thickening [32]. However, it is unclear whether the patients were symptomatic for cholecystitis. Hypoalbuminemia or capillary leak secondary to endothelial dysfunction might have contributed to gallbladder wall thickening in the absence of cholecystitis. Cases of calculous cholecystitis in the setting of HFRS have been reported in the literature [33,34]. Liu et al. reported a case of concomitant acute pancreatitis and acute capillary cholangitis in a patient with HFRS. The diagnosis of acute capillary cholangitis was based on cholestatic hepatic biochemistry, in the absence of biliary obstruction, and the exclusion of alternate causes [35].

Pancreatic Involvement

Acute pancreatitis is the most frequently reported form of pancreatic involvement in patients with hantavirus infection. Pancreatitis is mostly reported in patients with HFRS. In a meta-analysis by Ye et al., involving 11 studies and 1218 HFRS patients, the reported overall incidence of acute pancreatitis was 8.5% (95% CI: 5.9%-11.1%; Z = 6.40, p = 0.001). Additionally, acute pancreatitis was associated with increased mortality in patients with HFRS (OR = 3.668, 95% CI for OR: 1.112-12.031) [36]. Wang et al. compared patients with HFRS-associated acute pancreatitis and those with acute biliary pancreatitis over a six-year period. Symptoms did not differ between the two groups; however, bleeding manifestations, renal failure, and hepatic dysfunction were more common in patients with acute pancreatitis and in patients with HFRS. Patients with HFRS-associated acute pancreatitis had more severe thrombocytopenia and coagulopathy, which might have contributed to hemorrhagic manifestations [37]. The summary of pancreatic disease cases identified in a PubMed search is presented in Table 2 [7,35,38-44]. The diagnosis of pancreatitis requires two of the following three criteria: (a) characteristic abdominal pain; (b) elevated amylase and/or lipase, ≥3 times the upper limit of normal; and (c) imaging features. In patients with renal failure, pancreatic enzyme levels may be falsely elevated because of reduced renal clearance, making diagnosis challenging in the absence of abdominal imaging. 

**Table 2:** A summary of the reported cases of pancreatitis in hantavirus infection HFRS: hemorrhagic fever renal failure syndrome; HPS: hantavirus pulmonary syndrome; CT: computed tomography; RRT: renal replacement therapy.

Study	Age/Gender	Diagnosis of Pancreatitis	Associated Findings	Additional Interventions	Outcome
Dzagurova et al. (2019), 3 cases [7]	23 years; Male HFRS Sochi virus	Abdominal pain, ultrasound findings	Renal failure, multifocal pneumonia	Details not available	Discharge
	50 years; Male HFRS Sochi virus	Abdominal pain, ultrasound findings	Renal failure, pulmonary edema	Details not available	Discharge
	31 years; Male HFRS Sochi virus	Abdominal pain, ultrasound findings	Renal failure	Details not available	Discharge
Liu et al. (2022) [35]	34 years; Male HFRS	Abdominal pain, elevated amylase, lipase, CT: swollen pancreas with peripancreatic fluid, MRCP: no obstruction or stones	Renal failure, transaminitis, hyperbilirubinemia	Continuous RRT	Discharge
Yang et al. (2024) [38]	30 years; Male HFRS	Abdominal pain, normal amylase, lipase, CT: peripancreatic fluid	Renal failure, pulmonary edema, transaminitis	High-flow nasal cannula Hemodiafiltration	Discharge
Fan et al. (2013), case series: 12 cases [39]	38 ± 19 years Male: Female 9:3 HFRS	Abdominal pain (all patients). Amylase and lipase were elevated (all patients). CT was done in 10 patients: Enlarged pancreas and peripancreatic fluid in all 10 patients. Two of them had evidence of pancreatic necrosis	Renal failure (all patients), pulmonary edema (3 patients)	Dialysis x 4 Necrosectomy x 1	Death x 1`
Puca et al. (2012) [40]	53 years; Male HFRS Dobrava virus	Abdominal pain, elevated amylase, and lipase. CT: edematous pancreas, peripancreatic fluid	Renal failure, transaminitis	Nil	Discharage
Zhong et al. (2024) [41]	20 years; Male HFRS Hantaan virus	Abdominal pain, elevated amylase, and lipase. CT: pancreatic swelling, peripancreatic exudate	Renal failure, hypoxic respiratory failure, transaminitis, hyperbilirubinemia	High-flow nasal cannula; intermittent RRT	Discharge
Kilit et al. (2017) [42]	44 years; Male HFRS	Abdominal pain, elevated amylase, and lipase. CT: pancreas and peripancreatic edema	Renal failure, transaminitis	Hemodilaysis	Discharge``
Muco et al. (2020) [43]	45 years; Male HFRS	Abdominal pain, elevated amylase, and lipase	Renal failure, seizures, transaminitis	Nil	Discharge`
Kang et al. (2005), 2 cases [44]	41 years; Male HFRS Hantaan virus	Abdominal pain, elevated amylase, and lipase. CT: mild swelling in the pancreatic body and tail	Renal failure, transaminitis	Nil	Discharge
	70 years; Male HFRS Hantaan virus	Abdominal pain, elevated amylase and lipase, CT: edematous pancreatic body and tail	Renal failure, transaminitis	Nil	Discharge

**Figure 1:**
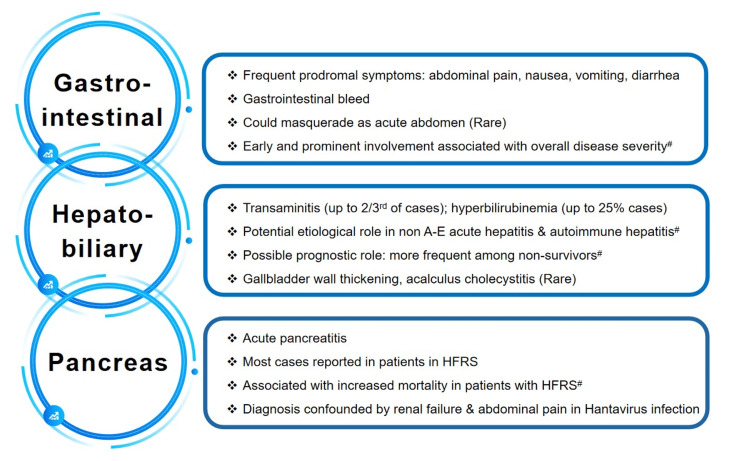
A summary of gastrointestinal and hepatic-biliary-pancreatic manifestations of hantavirus infection HFRS: hemorrhagic fever renal failure syndrome. ^#^Evidence is limited. Image generated using WPS Office (Kingsoft Office Software, Beijing, China).

In summary, GI symptoms are frequently reported during hantavirus infection and tend to precede organ-specific sequelae. Transaminitis and, less frequently, hyperbilirubinemia constitute the hepatobiliary manifestations. Gallbladder wall thickening of unclear significance, calculus cholecystitis, pancreatitis, and being a trigger for autoimmune hepatitis complete the spectrum of GI, hepatobiliary, and pancreatic implications of hantavirus infection. Figure 1 summarizes the entire spectrum of hantavirus syndrome from a gastroenterology perspective. 

Diagnosis

Early diagnosis of hantavirus infection is often challenging outside endemic regions, owing to the rarity of the disease and clinical features that overlap with most viral or flu-like syndromes. However, a high index of suspicion is indicated in patients with recent known or suspected exposure to rodents, travel to endemic regions, or contact with/exposure to confirmed cases, especially in those presenting with one or more of the following: fever, myalgias, GI or respiratory symptoms, renal dysfunction, or hemorrhagic manifestations [2].

Nonspecific laboratory derangements

The hematologic and biochemical profile provides the most reliable early diagnostic clues. Although nonspecific, common laboratory aberrations include thrombocytopenia, leukocytosis, elevated blood urea nitrogen and creatinine, and transaminitis. A review of all HPS cases reported in Texas from 1993 to 2006 confirmed this pattern, with thrombocytopenia in 92%, elevated creatinine in 61%, increased band forms in 52%, and hematocrit above 55% in 32% [15]. Thrombocytopenia occurs in 90% of patients with Puumala hantavirus infection, with platelet counts below 50 × 10⁹/L in 28% of cases. The severity of thrombocytopenia correlated with systemic inflammation and capillary leakage rather than the degree of renal injury [45]. Leukocytosis with a left shift, hemoconcentration, immunoblasts, and atypical lymphocytes are additional early findings [11]. In a retrospective cohort of 395 HFRS patients, 27.3% presented with disseminated intravascular coagulation on admission, and prolonged prothrombin time, low fibrinogen, and elevated total bilirubin were independently associated with mortality [24].

Specific diagnostic tests

Definitive diagnosis relies on the demonstration of specific antibodies to hantaviruses on serology or on the detection of viral nucleic acid by PCR. Serology via enzyme immunoassay is the first-line test; IgM antibodies are typically detectable at the onset of febrile illness, while IgG antibodies appear by the end of the febrile prodrome. Specific IgM immunochromatographic tests aid in identifying the specific hantavirus strains. IgM antibodies are diagnostic of an acute infection, whereas IgG antibodies can persist long after an acute infection. Cross-reactivity of antibodies with Epstein-Barr virus has been reported [46]. Reverse transcriptase PCR (RT-PCR) enables the diagnosis and speciation of hantaviruses with high sensitivity and specificity, even before symptom onset or the appearance of antibodies in serum [10]. The preferred sample for RT-PCR is whole blood. The buffy coat can be used for PCR, as hantaviruses, especially Andes virus, demonstrate tropism to mononuclear cells [47]. As the yield of a PCR test depends on viral load, the WHO interim guidance (2026) recommends repeating RT-PCR after 24 to 48 hours in cases where suspicion is high and the initial test is negative [46]. Genomic sequencing allows species-specific diagnosis and supports epidemiological evaluation. Viral cultures are technically demanding and may pose biohazard risks; therefore, they are not routinely recommended [46]. Patients with hantavirus syndromes should receive routine hemograms and organ-specific biochemical test batteries to assess disease involvement and guide treatment planning; tracking the results would improve analysis of disease progression and prognosis.

WHO 2026 case definitions

During the 2026 cruise ship outbreak, the WHO adopted four operational case definitions: (a) confirmed case, (b) not a case, (c) probable case, and (d) suspected case. Confirmed cases refer to patients proven to have an infection, while "not a case" refers to patients proven not to have an infection by RT-PCR or serology. Suspected cases were those exposed to a confirmed or probable case and having compatible symptoms: fever, myalgia, chills, acute GI (e.g., nausea, vomiting, diarrhea, abdominal pain), or acute respiratory (e.g., cough, dyspnea, chest pain) manifestations. Probable cases included patients with exposure and the aforementioned symptoms who did not undergo testing [5].

Treatment

There are no specific therapeutic agents available for the management of hantavirus infections. Current treatment is primarily symptomatic, coupled with organ-specific support as indicated. Close monitoring and correction of fluid and electrolyte status are essential in all patients. Patients with HFRS should be closely monitored for renal dysfunction; renal replacement therapy may be required in around 5% of patients [47,48]. Patients with severe HPS with hypoxic respiratory failure and acute respiratory distress syndrome will require mechanical ventilation or extracorporeal membrane oxygenation (ECMO). ECMO was associated with overall survival rates of 66.6% and 60.5% according to Wernly et al. and Dietl et al., respectively [49,50]. Wernly et al. compared an early ECMO strategy against initial ventilation, followed by ECMO, and reported better survival with early ECMO (80% vs. 56%; p = 0.048) [49]. A Chilean observational study reported improved survival among patients with Andes virus HPS who were referred early to an ECMO center; 82% survived among those who received ECMO [51]. Overall, the literature recommends early referral to ECMO centers for patients with HPS.

In a study from China involving patients with hantavirus HFRS syndrome, intravenous ribavirin was associated with a mortality benefit; however, subsequent studies in HFRS and HPS failed to replicate this effect [52-54]. In animal model studies, favipiravir, when administered early, before the onset of viremia, was associated with decreased viremia and reduced development of HPS with Andes and Sin Nombre viruses [55]. An in vitro study on Vero E-6 cells using Hantaan virus also demonstrated antiviral effects of ribavirin and favipiravir, as well as synergistic effects when combined [56]. There are currently no human studies to suggest a benefit of favipiravir in hantavirus infections. High-dose intravenous steroids were evaluated in an attempt to curtail possible overly active or dysregulated immune responses leading to HPS, but Vial et al. met with no clinically significant benefits [10].

Limitations

The narrative methodology used to prepare the manuscript may have introduced selection bias. As the current manuscript is synthesized from the published literature, publication bias is also likely, since uncommon manifestations tend to be reported or published more often than common ones. Incomplete information in the published literature might have precluded the data synthesis during manuscript preparation. The missing data from non-English publications, especially for a disease with high prevalence in China, where up to one-third of medical publications are in Chinese, might have contributed to analytical errors. The heterogeneity in the disease spectrum and the limited data on GI, hepatobiliary, and pancreatic manifestations of hantavirus infection from case reports, small case series, and observational studies further limit the strength and generalizability of conclusions.

## Conclusions

GI manifestations are among the most frequently reported features of hantavirus infection and often precede organ-specific disease. Hepatobiliary involvement typically manifests as transient elevations in transaminases and, occasionally, hyperbilirubinemia. Infrequently, gallbladder wall thickening, acalculous cholecystitis, and pancreatitis have been reported. Rare instances of hantavirus infection triggering autoimmune hepatitis have also been reported. Early and severe GI and hepatobiliary manifestations have been associated with more severe disease and worse outcomes; however, further clarification is required from large-scale prospective studies. It is unclear whether these manifestations reflect systemic endothelial dysfunction, a direct cytotoxic effect, or immune-mediated dysfunction.
